# Suidae Coronaviruses: Epidemiology, Transmission, and Molecular Diagnosis

**DOI:** 10.3390/ani16020257

**Published:** 2026-01-15

**Authors:** Chiara Ortello, Lorenzo Pace, Donatella Farina, Viviana Manzulli, Valeria Rondinone, Dora Cipolletta, Domenico Galante

**Affiliations:** 1 PhD National Programme in One Health Approaches to Infectious Diseases and Life Science Research, Department of Public Health, Experimental and Forensic Medicine, University of Pavia, 27100 Pavia, Italy; chiara.ortello01@universitadipavia.it; 2 Istituto Zooprofilattico Sperimentale della Puglia e della Basilicata, 71121 Foggia, Italy; donatella.farina@izspb.it (D.F.); viviana.manzulli@izspb.it (V.M.); valeria.rondinone@izspb.it (V.R.); dora.cipolletta@izspb.it (D.C.); domenico.galante@izspb.it (D.G.)

**Keywords:** swine coronaviruses, epidemiology, One Health

## Abstract

The emergence and spread of swine coronaviruses represent a growing challenge for both veterinary medicine and public health. These viruses exhibit high mutation rates, recombination potential, and the capacity for cross-species transmission. Among the most relevant pathogens are PEDV, TGEV, PRCV, PHEV, PDCoV, and SADS-CoV, which have caused significant outbreaks in swine production systems worldwide, with severe economic consequences. Recent evidence demonstrates coronavirus circulation in wild boar populations across Europe, including Italy, Spain, and Germany. Although wild boars are not confirmed as primary reservoirs, their ecological behavior and increasing overlap with domestic pigs raise concern over their potential role in maintaining viral circulation. Future research priorities should focus on developing a more integrated and coordinated system for the control of swine coronaviruses, including strengthened surveillance in both domestic pigs and wild boar populations, the use of molecular epidemiology techniques to identify emerging variants, and structured collaboration among veterinary, ecological, health, and regulatory sectors. Finally, investment is needed in the development of next-generation vaccines and diagnostic tools to address the considerable genetic variability of swine coronaviruses and to improve the prevention and early detection of and response to future epidemic threats.

## Simple Summary

Coronaviruses in domestic pigs and wild boars are a major concern for animal and public health. These viruses are characterized by large genomes, high mutation rates, and a remarkable ability to adapt to new hosts and environments. While historically considered of limited relevance, porcine coronaviruses such as Porcine Epidemic Diarrhea Virus, Transmissible Gastroenteritis Virus, Porcine Respiratory Coronavirus, Porcine Hemagglutinating Encephalomyelitis Virus, Porcine Deltacoronavirus, and Swine Acute Diarrhea Syndrome Coronavirus now represent significant threats. Recent studies have identified coronavirus antibodies and RNA in wild boars in Italy, Spain, Germany, and Poland, raising concerns over their role as reservoirs or amplifiers of infection. The One Health perspective underscores the need for integrated surveillance, improved diagnostic tools, and strategies to mitigate zoonotic risk.

## 1. Introduction

**Figure 1:**
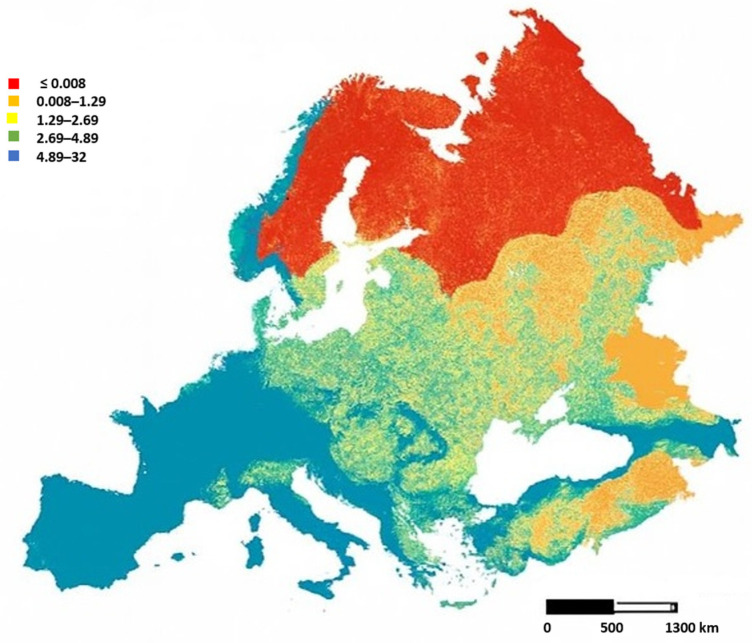
Map showing estimates of wild boar (*Sus scrofa*) distribution and abundance based on EFSA report 2024. Data expressed as individuals/km^2^.

The appearance and evolution of coronaviruses in domestic pigs (*Sus scrofa domesticus*) and wild boars (*Sus scrofa*) are a major concern for both animal health and public health due to their considerable potential zoonotic risk. They are characterized by large genomes (~27–32 kb), a high mutation rate, and frequent recombination events that enable rapid adaptation to new hosts and environments [1,2]. Coronaviruses (CoVs) have exhibited a penchant for jumping species barriers throughout history, with devastating effects. Historically, animal coronaviruses were considered of little importance to public health and global economic systems [3]. However, the recent emergence of highly pathogenic human coronaviruses, such as Severe Acute Respiratory Syndrome Coronavirus (SARS-CoV) in 2002–2003, Middle Eastern Respiratory Syndrome Coronavirus (MERS-CoV) in 2012, and SARS-CoV-2 in 2019, has fundamentally changed our view of the potential zoonotic risk of circulating coronaviruses in animals [3,4]. The importance of porcine coronaviruses is not limited to their impact on livestock health and economics. Due to their relatively high mutation rates, broad recombination potential, and adaptability, these viruses have demonstrated a high potential for zoonotic transmission [2]. Although human-to-human transmission has not been confirmed for these swine-origin coronaviruses, the risk of cross-species transmission remains significant, particularly in regions with a high animal density, limited surveillance, or poor sanitary conditions. The role of wild boars in the ecology of porcine coronaviruses remains poorly studied. However, recent studies have detected antibodies in wild boar sera against certain coronaviruses, such as Porcine Epidemic Diarrhea Virus and Transmissible Gastroenteritis Virus (PEDV and TGEV), suggesting past exposure and potential circulation in wild populations [5]. For these reasons, wild boars (*Sus scrofa*), as potential reservoirs or fallout hosts for porcine coronaviruses, have attracted increasing attention for their expansion in Europe, because this has increased the likelihood of contact with domestic pigs (Figure 1) [6]. All of this means that wild boars may occasionally contract infections from domestic herds, but may not serve as the main reservoirs. However, given the adaptability of coronaviruses, the role of wild suids cannot be underestimated in long-term viral ecology. The risk of recombination events between viruses circulating in wild boars and those in domestic pigs cannot be excluded [7]. Pigs, being susceptible to both avian and mammalian viruses, may act as potential intermediate hosts or amplifiers for novel recombinant viruses with zoonotic potential. Understanding the evolution, transmission, and ecology of swine coronaviruses thus requires a multidisciplinary approach that combines molecular biology, epidemiology, virology, and ecology. Advances in next-generation sequencing (NGS), metagenomics, and molecular diagnostics have enhanced the ability to detect, characterize, and monitor these viruses. However, significant gaps remain in our knowledge, especially regarding the role of wildlife in viral maintenance, the molecular mechanisms underlying cross-species transmission, and the development of effective vaccines and control strategies [7,8]. Given the close relationship between pigs, wild boars, and humans in many regions of the world, understanding coronavirus diversity and evolution in these hosts is essential for assessing zoonotic risk and preparing surveillance strategies [2,4,9]. This review paper summarizes the current state of knowledge about swine coronaviruses, focusing on epidemiology, diagnostic techniques, surveillance strategies, and the One Health approach.

## 2. Classification of Swine Coronaviruses

Coronaviruses (order *Nidovirales*, family *Coronaviridae*, subfamily *Coronavirinae*) are enveloped, positive-stranded RNA viruses that, according to their genetic characteristics, are divided into four genera: *Alphacoronavirus*, *Betacoronavirus*, *Gammacoronavirus*, and *Deltacoronavirus* [10]. *Alphacoronaviruses* and *Betacoronaviruses* are found exclusively in mammals, whereas *Gammacoronaviruses* and *Deltacoronaviruses* primarily infect birds. The evolutionary origin of many swine coronaviruses can be traced back to wildlife reservoirs, particularly for bats and birds, which serve as major hosts for *Alpha-* and *Deltacoronaviruses*, respectively [11]. Swine are susceptible to several species of coronaviruses. Among the most common are Porcine Epidemic Diarrhea Virus (PEDV), Transmissible Gastroenteritis Virus (TGEV), Porcine Respiratory Coronavirus (PRCV), Hemagglutinating Encephalomyelitis Virus (PHEV), Porcine Deltacoronavirus (PDCoV), and Swine Acute Diarrhea Syndrome Coronavirus (SADS-CoV). Each of these pathogens has distinct epidemiological and pathological features (Table 1) but collectively pose a constant threat to swine production systems worldwide [12,13], especially in countries where biosecurity practices are limited [14]. The importance of swine coronaviruses is not only limited to their impact on livestock health and economics, but these viruses have demonstrated a high potential for zoonotic transmission. In particular, PDCoV and SADS-CoV have demonstrated the ability to infect human cell lines in vitro and have been detected in febrile human patients [13,15]. Swine coronaviruses are globally distributed, with PEDV and PDCoV reported in Asia, North America, and parts of Europe. The spread of these viruses has been facilitated by swine trade, globalized agriculture, and contaminated feed and equipment [16]. The role of wild boars (*Sus Scrofa*) in the ecology of swine coronaviruses remains under-investigated. However, serological investigations in wild boars have reported antibodies against TGEV, PEDV, and PRCV, although evidence of sustained viral circulation remains scarce [17]. Despite limited data, serological and molecular evidence suggests that wild boars may harbor swine CoVs or related strains. Their potential role as mixing vessels or spillback hosts remains underexplored but is plausible, especially in regions where wild boars interact with domestic swine [18]. The genetic plasticity of coronaviruses, particularly in the spike (S) glycoprotein, facilitates cross-species transmission and the emergence of novel variants [19]. This is exemplified by the emergence of SADS-CoV, from bat-to-pig transmission, and the recurrent emergence of PEDV variants with altered pathogenicity. SADS-CoV has been linked to bat coronaviruses of the HKU2 lineage, while PDCoV likely originated from avian CoVs [20]. Phylogenetic analyses suggest that multiple historical and recent cross-species transmission events have contributed to the emergence of swine CoVs, some of which may pose future zoonotic risks. Swine CoVs exhibit high mutation rates due to the lack of proofreading in their RNA-dependent RNA polymerases. However, uniquely among RNA viruses, coronaviruses encode an exonuclease (nsp14) that provides limited proofreading, balancing genome stability with adaptability [21]. Their long genomes (~27–32 kb) allow space for accessory genes and recombination hotspots, especially in the spike (S) protein gene, which plays a crucial role in host tropism and immune evasion [19]. For example, PEDV strains show considerable diversity. Classic strains (G1s) differ antigenically and genetically from the highly pathogenic variants (G2s) responsible for large-scale epidemics in Asia and North America [16]. PRCV is believed to have originated from a deletion in the S gene of TGEV, which altered tissue tropism from the gastrointestinal tract to the respiratory tract [22]. Despite this change, the two viruses share over 96% genomic similarity, demonstrating the role of small mutations in altering the dynamics and epidemiology of the disease [23]. The emergence of swine enteric coronavirus (SeCoV) in Europe demonstrates the threat posed by recombination. SeCoV is a natural recombinant of PEDV and TGEV identified in Germany and Italy that retains genetic regions from both parental viruses, indicating ongoing viral evolution in the swine population [24]. Such recombinants may escape diagnostic tests, evade pre-existing immunity, or acquire new pathogenic properties. Molecular epidemiology studies using next-generation sequencing (NGS) and Bayesian phylogenetics have traced the origins and spread of PEDV and PDCoV strains across Asia, North America, and Europe. For example, the G2b PEDV strain likely originated in China before entering the U.S. in 2013 [25]. European strains are more genetically conservative, but recombination events with Asian strains have been detected, especially in Italy and Germany [24]. PDCoV shows similarity to avian coronaviruses and has been reported in multiple continents [26]. Despite limited data, serological and molecular evidence suggests that wild boars may harbor swine CoVs or related strains. Their potential role as mixing vessels or spillback hosts remains underexplored but is plausible, especially in regions where wild boars interact with domestic swine [18]. The factors that may facilitate the spread and evolution of swine coronaviruses include the following:

- High animal density: increases chances of co-infection and recombination;
- Intensive pig farming: amplifies viral transmission and mutation pressure;
- Global trade: facilitates the spread of the virus across different territories;
- Wildlife interfaces: enable spillover and adaptation to new hosts.

Swine coronaviruses exhibit remarkable genetic plasticity and adaptive potential. Their evolution is shaped by recombination, mutation, host-switching, and anthropogenic factors. Genomic surveillance, particularly using NGS and metagenomics, is essential to monitor the emergence of novel strains, understand evolutionary dynamics, and inform vaccine design and public health preparedness.

### 2.1. Porcine Epidemic Diarrhea Virus

Porcine Epidemic Diarrhea Virus (PEDV) has been one of the most detrimental coronaviruses for pig farming in the 21st century. After first being identified in the UK in 1971, PEDV became endemic in Asia and caused devastating outbreaks in China in 2010–2011 and in the United States in 2013, with piglet mortality rates exceeding 90% in some herds [25,31]. In Europe, PEDV circulation has been relatively limited since the 1990s, though localized outbreaks were reported in Germany, Italy, and Eastern Europe after 2014 [24,45]. Italian outbreaks were linked to strains genetically related to the U.S. and Asian lineages, confirming viral flow [24]. PEDV is a highly contagious enteric coronavirus that causes severe enteritis in swine, particularly in neonatal piglets, leading to significant morbidity, mortality, and economic losses worldwide. In the last two decades, the emergence of highly virulent strains, particularly in Asia and North America, has made PEDV a major threat to global swine production [14,46]. PEDV belongs to the genus *Alphacoronavirus*, family *Coronaviridae*, order *Nidovirales*. It shares genomic and structural similarities with Transmissible Gastroenteritis Virus (TGEV) and feline coronavirus but possesses distinct epidemiological and pathological features. PEDV primarily infects and destroys mature enterocytes lining the small intestine, resulting in villous atrophy, malabsorption, and osmotic diarrhea. In neonatal piglets, their immature immune system and underdeveloped gut render them particularly susceptible. Clinical signs include profuse watery diarrhea, vomiting, lethargy, severe dehydration, and high mortality in piglets. In older pigs and sows, the infection tends to be self-limiting but still causes weight loss and reproductive impact due to sow anorexia and lactation failure [25,31,47]. Adult pigs may experience milder diarrhea but still act as important reservoirs. The PEDV genome is approximately 28 kb in length and encodes several structural proteins—spike (S), envelope (E), membrane (M), and nucleocapsid (N)—as well as non-structural proteins involved in replication and transcription [48]. Molecular studies classify PEDV strains into two genogroups:

- G1s (classical strains): Originally reported in Europe and Asia in the 1970s–1990s.
- G2s (highly pathogenic strains): Emerged in China in 2010 and were responsible for major outbreaks in the U.S. in 2013.

Subtypes such as G1a, G1b (S-INDEL), G2a, and G2b have been proposed based on full genome sequencing and phylogenetic analyses [16].

PEDV is primarily transmitted via the fecal–oral route through contact with infected feces or vomitus, either directly between pigs or indirectly via contaminated environments, feed, or equipment. Fecal shedding begins 1–2 days post-infection and lasts 7–10 days, occasionally up to 36 weeks [32,33]. Its high viral load and environmental stability, especially at low temperatures, facilitate spread. PEDV has mainly affected Asia, North America, and parts of Europe. Highly virulent strains emerged in China around 2010, causing major outbreaks in South Korea, Vietnam, Japan, Thailand, and the Philippines [25]. In 2013, PEDV appeared in the U.S., killing over 7 million piglets and later spreading to Canada and Mexico. In Europe, S-INDEL strains reemerged between 2014 and 2016, causing generally milder outbreaks, likely due to lower virulence, partial herd immunity, or improved biosecurity. Italy reported G1b S-INDEL variants in 2014–2015, with sporadic cases mainly in northern regions like Emilia-Romagna and Lombardia [49]. Serological surveys detected PEDV antibodies in 3.83% of wild boars in Italy and 3.2% in Poland, suggesting past exposure, while active viral RNA was identified in 9.75% of South Korean wild boars [34,50,51]. Transmission to wild boars may occur through contaminated feed or water, indirect contact with domestic pigs [52], or human activities like hunting. Though not primary reservoirs, wild boars can act as subclinical carriers or sentinels, highlighting the need for ongoing surveillance and biosecurity, especially where wild boar populations overlap with pig farms [53,54,55].

### 2.2. Transmissible Gastroenteritis and Porcine Respiratory Coronavirus

Transmissible Gastroenteritis Virus (TGEV) is an *Alphacoronavirus* of swine first described in the 1940s in the United States. It is a highly contagious enteric pathogen that historically caused devastating outbreaks characterized by severe diarrhea, dehydration, and up to 100% mortality in neonatal piglets [27]. Although its global prevalence has markedly declined since the late 1980s, TGEV remains important as a model for coronavirus pathogenesis and evolution [23].

Porcine Respiratory Coronavirus (PRCV) emerged in the 1980s as a natural deletion mutant of TGEV, resulting from a large deletion (~681 nucleotides) in the 5′region of the spike (S) gene. This genetic change led to a shift in tissue tropism from the intestinal epithelium to the respiratory tract. While TGEV causes severe enteric disease, PRCV typically induces mild or subclinical respiratory infections with low mortality [22,23].

Both viruses belong to the genus Alphacoronavirus and share over 96% genome sequence similarity. Their genomes encode four structural proteins (S, E, M, and N), replicase polyproteins (ORF1a and ORF1b), and accessory proteins (ORF3a and ORF3b) [56]. Despite their genetic similarity, differences in the S protein account for distinct pathogenicity and transmission routes [22]. TGEV is primarily transmitted via the fecal–oral route, through contaminated feces, feed, water, and fomites, although respiratory transmission has also been demonstrated. In contrast, PRCV spreads mainly via aerosols and respiratory secretions, with minimal or absent fecal shedding, facilitating efficient dissemination within and between herds [28,57,58,59]. Indirect transmission through fomites, including contaminated equipment, feed, clothing, and vehicles, has been documented as well [30].

The widespread circulation of PRCV has contributed to the decline in TGEV by inducing cross-protective immunity in pig populations. Consequently, vaccination against TGEV and strict biosecurity measures remain the main control strategies, with colostrum-derived maternal immunity being critical for piglet protection. PRCV has no specific vaccines, but its endemic presence acts as a form of natural immunization [29].

Serological surveys indicate that PRCV is endemic in most pig-producing regions worldwide, including Europe, the United States, and Asia. In Italy, TGEV has been reported since the 1960s but has declined significantly since the late 1990s, whereas PRCV remains widely prevalent. Wild boars have shown serological evidence of exposure to both viruses, suggesting a potential reservoir role and a risk for re-emergence in naïve domestic herds [60,61].

Although TGEV and PRCV have not demonstrated zoonotic potential, their evolutionary relationship highlights the adaptability of coronaviruses. Continued surveillance, particularly using molecular and genomic tools, is essential to detect potential re-emergence, recombination events, or the emergence of atypical strains.

### 2.3. Porcine Hemagglutinating Encephalomyelitis Virus

Porcine Hemagglutinating Encephalomyelitis Virus (PHEV) is a neurotropic coronavirus belonging to the *Coronaviridae* family, *Betacoronavirus* genus, *Embecovirus* subgenus [35]. It is the causative agent of Porcine Hemagglutinating Encephalomyelitis (PHE), historically known as “vomiting and wasting disease” (VWD) due to its gastrointestinal signs [36] and as “hemagglutinating encephalomyelitis” when neurological signs dominate [37]. PHEV primarily infects pigs less than 4 weeks of age, resulting in high morbidity and variable mortality, while older animals typically remain asymptomatic due to previous exposure and immunity. High mortality occurs if piglets become infected before maternal antibodies decrease [36]. First described in Canada in 1957, PHEV has since been reported on several continents, including Asia, North America, and Europe. Despite its wide distribution, the virus is considered sporadic, with epidemics occurring rarely but potentially leading to significant losses on farms [35]. Unlike other porcine coronaviruses, such as PEDV, TGEV, or PDCoV, PHEV is not primarily enteropathogenic but neurotropic and its clinical impact is closely related to the age of the pig at the time of infection and the immune status of the sow herd [62]. PHEV is an enveloped, positive-sense, single-stranded RNA virus with a genome of approximately 30 kb [35]. The virus exhibits hemagglutinating properties due to the hemagglutinin esterase (HE) protein, which distinguishes Embecoviruses from other coronaviruses [63]. The HE protein contributes to receptor binding and neuroinvasion, acting synergistically with the viral spike (S) protein, which binds sialic acid residues [64]. Porcine Hemagglutinating Encephalomyelitis Virus (PHEV) spreads primarily through direct contact between pigs, especially in breeding herds where the virus can circulate silently. The respiratory route represents the main pathway of infection: viral replication begins in the nasal mucosa, tonsils, and lungs, and transmission occurs via respiratory droplets and aerosols shed by infected pigs [35]. Indirect transmission is also possible through contaminated fomites, such as equipment, boots, or clothing, highlighting the importance of strict biosecurity measures to limit farm-to-farm spread. The role of lactogenic immunity is critical in the epidemiology of PHEV. Piglets from immune sows acquire protective antibodies via colostrum and milk, which prevent disease even if exposure occurs. In contrast, piglets from seronegative sows, or those that lose maternal immunity too early, are highly susceptible to infection [65]. After oral or nasal infection, PHEV replicates initially in the upper respiratory tract and tonsils, before spreading via peripheral nerves, particularly the trigeminal and vagus nerves, to the CNS [64]. This explains the dual syndromes: vomiting/wasting (via vagus nerve involvement) and encephalomyelitis (via CNS replication). Globally, PHEV has been documented in Canada, the United States, Brazil, China, Japan, South Korea, and several European countries [35,66]. Most reports describe sporadic outbreaks rather than continuous endemic circulation. In Europe, reports exist from Germany, the Netherlands, and the UK in the mid–late 20th century [36,37]. In Italy, PHEV has not been extensively studied compared with PEDV or TGEV, and, to date, no confirmed clinical cases of PHEV in wild boars have been reported in Europe. Given the overlap between wild and domestic suids, especially in Italy, where wild boar populations are expanding, surveillance is strongly recommended [38] and is mostly passive, with occasional serological surveys. Control measures rely on maintaining herd immunity [36], biosecurity against viral introduction, and monitoring wildlife as a possible reservoir [38]. Comparative genomic studies have revealed that PHEV is closely related to human coronaviruses OC43 and HKU1, as well as bovine coronavirus (BCoV). This phylogenetic proximity suggests a history of interspecies transmission and adaptation, further underscoring the zoonotic relevance of PHEV [67,68]. This evolutionary link underscores potential interspecies transmission events [38]. Although no zoonotic infections have been reported, the inclusion of wild boars in surveillance aligns with the One Health framework.

### 2.4. Swine Acute Diarrhea Syndrome Coronavirus and Porcine Deltacoronavirus

Pig farms continue to face significant challenges due to the emergence and re-emergence of enteric coronaviruses, in particular, Porcine Acute Diarrhea Syndrome Coronavirus (SADS-CoV) and Porcine Deltacoronavirus (PDCoV), which have attracted global attention for their high pathogenicity in piglets and potential zoonotic risk. Coronavirus Acute Porcine Diarrhea Syndrome (SADS-CoV) was first identified in Guangdong, China, in 2016, and was linked to a severe outbreak of diarrhea in piglets with high mortality. SADS-CoV is an *Alphacoronavirus* closely related to bat coronavirus HKU2, and it shares ~98% genome identity with Rhinolophus bat CoVs detected in Guangdong province, China. Its genome is approximately 27.2 kb long and encodes standard coronavirus proteins. The spike protein of SADS-CoV lacks significant homology to known human coronaviruses but shares structural characteristics that allow efficient replication in a variety of mammalian cells. SADS-CoV outbreaks caused massive piglet mortality in affected farms, with clinical signs resembling PEDV and TGEV [20]. Experimental infections confirmed its ability to replicate efficiently in the small intestine, causing severe diarrhea and dehydration in neonatal pigs [33]. Although no zoonotic cases have been documented, its close relation to bat coronaviruses raises significant concern. To date, there are no reports outside China, but the close genetic relationship to bat coronaviruses and demonstrated replication in human cell lines underscore its pandemic potential [69]. The emergence of SADS-CoV highlights the porcine–bat interface as a critical hotspot for coronavirus spillovers. Porcine Deltacoronavirus (PDCoV), a *Deltacoronavirus* discovered in Hong Kong in 2012 through molecular surveillance, is an emerging enteric pathogen that has since been reported in North America, Asia, and more recently in Europe [26,43]. Its pathogenic role became evident in the U.S. in 2014, where it caused enteric disease outbreaks similar to PEDV. Since then, PDCoV has been reported in Canada, Mexico, South Korea, China, Thailand, Laos, and Vietnam [39]. In Europe, PDCoV has been detected sporadically, including molecular evidence in Italy suggesting silent circulation without major clinical outbreaks [24]. The genome is ~25.4 kb in size, smaller than PEDV or TGEV, and encodes a spike protein with moderate homology to avian deltacoronaviruses. Unlike PEDV and TGEV, PDCoV has been experimentally shown to cross the species barrier and infect calves and chickens, raising concerns about its zoonotic potential [70]. More concerning is PDCoV, which was isolated from blood samples of Haitian children with febrile illness [15]. Genomic analysis confirmed the presence of complete PDCoV genomes in these patients, and the virus showed evidence of replication in human cells. This represents the first confirmed case of swine deltacoronavirus infection in humans and suggests potential for future cross-species adaptation. For SADS-CoV, the predominant transmission route is the fecal–oral pathway, with infected piglets shedding high amounts of the virus in feces, contaminating the environment and facilitating rapid spread within herds [20,44]. Direct contact between pigs, especially between sows and neonatal piglets, plays a major role in farm-level outbreaks, while contaminated feed, water, and fomites may further support virus persistence and dissemination [42,71]. Environmental contamination, including contaminated surfaces and manure, has been reported as an important factor in sustaining transmission cycles [72]. Similarly, PDCoV is mainly transmitted via the fecal–oral route, with high viral loads detected in diarrheic feces of piglets [39,73]. Direct contact between animals accelerates farm-to-farm spread, while indirect transmission through contaminated equipment and poor biosecurity practices has been documented [40,41]. Although aerosol transmission has been hypothesized at short distances, fecal–oral exposure remains the most efficient pathway [74]. The frequent movement of pigs, coupled with inadequate sanitation, has been identified as a major risk factor for PDCoV outbreaks in Asia and North America [73,74]. SADS-CoV causes acute watery diarrhea, vomiting, and rapid dehydration, leading to high mortality rates (up to 90%) in piglets less than five days old [20,42]. Infected piglets show villous atrophy and extensive lesions in the small intestine, which compromise nutrient absorption and result in fatal outcomes [20]. Adult pigs can be infected but typically display mild or subclinical signs, serving as potential carriers within herds [72]. To date, SADS-CoV has not been reported in wild boars and there is no published evidence of infection. However, given the role of wildlife–livestock interfaces in coronavirus emergence, surveillance in wild boars has been recommended [75]. PDCoV causes acute enteric disease in piglets, with clinical signs including watery diarrhea, vomiting, dehydration, and reduced weight gain [12,76]. Mortality rates are usually lower than those caused by SADS-CoV or PEDV, but morbidity is high, especially in neonatal piglets [41]. Histologically, PDCoV infection leads to villous atrophy and epithelial damage in the small intestine, similar to other porcine enteric coronaviruses [77]. Unlike SADS-CoV, PDCoV has occasionally been investigated in wild boars. So far, there are no confirmed natural infections in wild boar populations, though serological surveys and molecular studies are limited. The virus has a broad experimental host range, infecting chickens, turkeys, and calves under experimental conditions [77,78], which suggests that wildlife species, including wild boars, could theoretically serve as reservoirs or spillover hosts. Nevertheless, all confirmed field cases remain restricted to domestic pigs. SADS-CoV and PDCoV represent emerging threats to swine health and potentially to human health. While their current distribution appears geographically limited, their demonstrated cross-species transmission potential, high mutation rates, and clinical impact on piglets justify heightened surveillance and research investment. In the context of One Health, the proactive monitoring of both viruses in domestic pigs, wild boars, and human populations is essential.

## 3. Molecular Diagnostic Techniques for Swine Coronaviruses

Rapid and accurate diagnosis is essential for effective disease management, epidemic control, and epidemiological surveillance. Molecular biology techniques have revolutionized veterinary diagnostics, offering speed, sensitivity, and specificity [79]. The CRISPR-based diagnostics reverse transcription polymerase chain reaction (RT-qPCR) remains the gold standard for the detection of porcine coronaviruses, although new molecular tools, such as isothermal amplification methods (LAMP, RPA), digital droplet PCR (ddPCR), and CRISPR-based diagnostics, are increasingly being used as complementary or alternative approaches. RT-qPCR is the main diagnostic tool to investigate cases of acute diarrhea, vomiting, and high mortality in newborn piglets, which are characteristic symptoms of PEDV, TGEV, PDCoV, or SADS-CoV. Veterinarians rely on RT-qPCR for rapid differentiation between these viruses, enabling personalized biosafety and management interventions [12,42]. The quantitative results obtained thanks to this method not only confirm the infection but also provide information on the viral load, which is particularly useful for monitoring the severity and progression of the disease [80]. The quantitative reverse transcription polymerase chain reaction (RT-qPCR) is the foundation of molecular diagnostics of porcine coronaviruses. It combines the reverse transcription of viral RNA into complementary DNA (cDNA) with quantitative PCR amplification, enabling real-time monitoring of the amplification process. This allows not only the detection but also the quantification of viral RNA in clinical and environmental samples. The diagnostic process begins with the extraction of high-quality RNA from feces, intestinal tissues, oral fluids, or environmental swabs. Reverse transcriptase enzymes are then used to synthesize cDNA from viral RNA templates. Subsequent PCR amplification is monitored by fluorescence-based chemicals, which allow the precise quantification of viral RNA loading [79]. Two major detection chemicals are commonly employed:

- SYBR Green assays based on the intercalation of fluorescent dye in double-stranded DNA. Although cost-effective, SYBR Green assays can suffer from nonspecific amplification and require post-amplification melting curve analysis.
- Assays based on TaqMan probes use fluorescently labeled probes that bind specifically to target sequences, providing greater specificity and enabling multiplexing capabilities [80].

RT-qPCR assays offer extremely high sensitivity and specificity, capable of detecting up to 10–100 copies of viral RNA per reaction. They are significantly more sensitive than the conventional techniques of RT-PCR, ELISA, or virus isolation [12,81]. Given the co-circulation of multiple porcine enteric coronaviruses, multiplex PCR assays have been developed to simultaneously detect PEDV, PDCoV, TGEV, and SADS-CoV in a single reaction [82]. Multiplex RT-qPCR reduces the reagent time, labor, and costs compared with singleplex assays. RT-qPCR is the reference method in both the diagnostic laboratories and epidemiological surveillance programs of porcine coronaviruses worldwide [75]. The first step in the development of RT-qPCR is the accurate design of primers and probes (Table 2). Target regions must be highly conserved within the viral genome but also unique enough to prevent cross-reactivity with other porcine pathogens or host genetic material. For coronaviruses, the nucleocapsid (N) gene and the spike (S) gene are frequently used targets because they are expressed in abundance and provide reliable sensitivity [81]. The action of RT-qPCR as a gold standard for coronavirus detection in pigs has transformed the way researchers, veterinarians, and surveillance authorities monitor and control these pathogens. In addition to its diagnostic utility in individual diseased animals, RT-qPCR has broad applications in surveillance programs, outbreak response, and epidemiological investigations, offering training in viral prevalence, transmission patterns, and strain evolution. During outbreaks, RT-qPCR serves as a rapid response tool to confirm etiological agents and guide immediate containment measures. For example, during the 2013 PEDV epidemic in the United States, RT-qPCR was widely used to confirm the presence of PEDV in pig farms in several states, allowing epidemiologists to monitor the rapid spread nationwide [75]. By analyzing diffusion dynamics in different age groups, researchers can estimate infectious dose, the duration of viral spread, and the likelihood of environmental persistence [81]. Although most diagnostic tests are focused on domestic pigs, RT-qPCR has also been applied to wild boar populations to assess the risk of coronavirus reservoirs and potential relapse events. Studies using RT-qPCR have detected the antibodies and, in some cases, viral RNA of porcine coronaviruses in wild boars in Europe, suggesting that they may contribute to the maintenance of the coronavirus at the wildlife–livestock interface [50].

## 4. Discussion

Emerging and re-emerging pathogens pose a persistent threat to both animal and human health. Among livestock species, pigs have increasingly been identified as major hosts for a wide range of viruses, many of which can establish long-term persistence. In addition, pigs act as reservoirs for several zoonotic agents, underscoring their importance at the human–animal interface [2]. Transmission of swine coronaviruses occurs predominantly via the fecal–oral route [75], a highly efficient mechanism that facilitates direct and also indirect transmission, by environmental contamination and subsequent infection through ingestion of contaminated water, food, or soil. In recent years, intensified interactions between wildlife, livestock, and human populations have further increased the risk of viral spillover events. Experimental studies demonstrate that some swine coronaviruses can infect Vero cells and certain bat species, suggesting potential intermediate host adaptation pathways [20]. Although in vitro studies indicate limited replication in human cells and no natural human infections have been reported to date, the continuous evolution of these viruses warrants sustained surveillance. A clear illustration of coronavirus evolution within swine is provided by transmissible gastroenteritis virus (TGEV) and porcine respiratory coronavirus (PRCV). The emergence of PRCV through gene deletion events from TGEV highlights how viral evolution can influence pathogenicity, immunity, and epidemiological dynamics. Understanding these processes is essential for developing effective control strategies. More broadly, constant surveillance is required not only for newly emerging viruses but also for known pathogens that may reappear after long periods of absence or exhibit altered characteristics, such as shifts in host range, prevalence, or geographic distribution.

In addition, in recent decades, several novel viral diseases have emerged in pig populations worldwide, emphasizing the complexity of porcine coronavirus epidemiology, particularly at the livestock–wildlife interface. Porcine epidemic diarrhea virus (PEDV) remains one of the most devastating swine coronaviruses, with severe outbreaks reported across Asia, North America, and Europe, and serological evidence suggesting spillover into wild boar populations [34]. TGEV and PRCV exemplify viral evolution through deletion events, with PRCV contributing to the decline in TGEV prevalence while simultaneously complicating surveillance and serological interpretation. Although porcine hemagglutinating encephalomyelitis virus (PHEV) occurs sporadically, it demonstrates the neurotropic potential of swine coronaviruses and the need to consider diverse pathogenic mechanisms [35]. More recently, swine acute diarrhea syndrome coronavirus (SADS-CoV) and porcine deltacoronavirus (PDCoV) have raised particular concern due to their phylogenetic relationships with bat and avian coronaviruses, broad host tropism, and experimental evidence suggesting zoonotic potential [15,20].

The role of wild boars in the epidemiology of swine coronaviruses remains unresolved. While serological surveys conducted in various European regions have detected antibodies against PEDV, TGEV, and PRCV, active viral replication or sustained circulation within wild boar populations has rarely been confirmed [86]. This discrepancy between serological evidence and direct viral detection complicates efforts to determine whether wild boars function as true reservoirs, incidental spillover hosts, or merely sentinels of environmental viral exposure. Despite these uncertainties, several ecological and anthropogenic drivers increase the likelihood of coronavirus persistence and the emergence of novel variants. High population densities of domestic pigs and wild boars, combined with habitat expansion, climate change, and the growing interconnectedness of global trade networks, create favorable conditions for viral dissemination. Inadequate or inconsistent biosecurity practices—particularly in outdoor or small-scale production systems—further amplify these risks by facilitating interactions at the wildlife–livestock–human interface [6]. Among enteric swine coronaviruses, PEDV is the most extensively studied, and vaccines are available, although their effectiveness under field conditions remains variable [14]. In contrast, substantially more research is needed for PDCoV and SADS-CoV to clarify their emergence, evolutionary trajectories, pathogenesis, and host immune responses. At present, disease prevention and control rely primarily on maternal immunity and farm-level management, with a strong emphasis on strict biosecurity and containment of infections within and between farms [75].

Rapid and accurate diagnosis is essential to prevent viral spread. Advances in molecular diagnostics, including RT-qPCR, droplet digital PCR, and CRISPR-based assays, have greatly improved the sensitivity and specificity of porcine coronavirus detection and monitoring [79,80]. Given the frequent co-circulation of multiple coronaviruses, multiplex PCR assays are increasingly valuable for simultaneous detection and enhanced outbreak response [82]. Nevertheless, significant knowledge gaps persist regarding viral evolution, cross-species transmission, and long-term maintenance in wildlife hosts.

Molecular epidemiology provides critical insights into the evolutionary dynamics of swine coronaviruses and their capacity to adapt to new hosts [87]. Phylogenetic analyses indicate that PEDV, PDCoV, and SADS-CoV have undergone multiple host-switching events, often involving wildlife reservoirs such as bats or birds [88]. Due to their susceptibility to both avian and mammalian viruses, pigs represent a unique ecological “mixing vessel” in which viral recombination and adaptation may facilitate cross-species transmission [88]. Transmission dynamics are increasingly shaped by human-mediated factors. International trade of live animals, animal feed, and farming equipment has repeatedly been implicated in long-distance viral dissemination. At the local scale, inadequate biosecurity, outdoor production systems, and contact with wildlife increase the risk of viral introduction and persistence. These observations indicate that traditional farm-level control measures, while necessary, are insufficient when implemented in isolation.

From a public health perspective, the risk posed by swine coronaviruses should be viewed probabilistically rather than deterministically. Although most porcine coronaviruses currently lack efficient human-to-human transmission, their demonstrated ability to infect human cells, coupled with high mutation rates and recombination potential, suggests a non-negligible risk over evolutionary time [89]. Continuous occupational exposure of humans working in pig production systems further increases opportunities for viral adaptation. Consequently, effective risk mitigation should prioritize early detection, reduction in viral circulation in animal populations, and minimization of cross-species contact. Surveillance strategies that integrate domestic pigs, wild boars, and human populations at elevated occupational risk would substantially enhance preparedness for future emerging coronaviruses [90].

## 5. Conclusions

Swine coronaviruses constitute a complex and evolving group of pathogens with significant implications for animal health, food security, and potentially public health. Their large genomes, high mutation rates, and recombination capacity enable rapid adaptation to changing ecological and epidemiological conditions. The emergence and spread of viruses such as PEDV, PDCoV, and SADS-CoV highlight how modern livestock production systems can function as amplification environments for novel coronaviruses with cross-species transmission potential [20]. Although wild boars are not currently confirmed as stable reservoirs [29,50], accumulating evidence indicates that they participate in the broader ecological network of swine coronaviruses. Their role as spillover hosts or epidemiological sentinels is particularly relevant in regions characterized by expanding wild boar populations and intensive pig farming. Ignoring this interface may result in missed opportunities for early detection and risk mitigation. Future control strategies must move beyond pathogen-specific approaches and adopt an integrated One Health framework. Strengthened and harmonized surveillance, coupled with molecular epidemiology tools such as next-generation sequencing and metagenomic analyses, will be essential to monitor viral evolution, detect recombination events, identify emerging variants, and clarify transmission pathways at the wildlife–livestock–human interface. Equally important is sustained investment in advanced diagnostics and research programs, broadly protective vaccines, and cross-sector collaboration among veterinary, public health, ecological, and regulatory authorities. In conclusion, proactive and integrated surveillance, informed by molecular and ecological data, represents the most effective strategy to reduce the impact of swine coronaviruses and to mitigate their potential role in future zoonotic events.

## Figures and Tables

**Table 1:** Main characteristics of swine coronaviruses.

Virus	Epidemiology	Target Organs	Main Symptoms	Transmission	References
TGEV	Major outbreaks in Europe, Asia, USA	Small intestine (enterocytes)	Severe watery diarrhea, vomiting, dehydration	Fecal–oral route, ingestion of contaminated feces, feed, or water	[23,24,27,28,29]
PRCV	Major outbreaks in Europe, Asia, USA	Respiratory tract (nasal mucosa, trachea, lungs)	Mild respiratory disease, coughing, nasal discharge	Via aerosols, respiratory secretions, and direct nasal–oral contact	[22,29,30]
PEDV	Major epidemics in USA, China, Korea; continues to circulate in Asia and parts of Europe	Small intestine (villus enterocytes)	Profuse watery diarrhea, vomiting, dehydration	Fecal–oral route, contaminated environments, feed, or equipment	[24,25,26,31,32,33,34]
PHEV	Reported in Asia, North America, and Europe	Central nervous system, stomach	Vomiting, wasting, ataxia, paralysis	Oro-nasal route, exposure to contaminated secretions	[35,36,37,38]
PDCoV	Reported in USA (2014); now detected in North America and Asia	Small intestine	Watery diarrhea, vomiting, dehydration	Fecal–oral route, contaminated equipment	[12,15,26,39,40,41]
SADS-CoV	Reported in North America, Asia, and more recently in Europe	Small intestine	Acute watery diarrhea, severe dehydration, high mortality	Fecal–oral route	[20,26,42,43,44]

**Table 2:** Molecular detection of main swine coronaviruses.

Virus	Primers/Probes	Sequences (5′-3′)	References
TGEV	Forward	GTGGTAATATGYTRTATGGCYTACAA	
	Reverse	GCCAGACCATTGATTTTCAAAACT	[83]
	Probe	TTGCTTATTTACATGGTGCYAGT	
PRCV	Forward	TTGTCTGGGTTGCCAAGGAT	
	Reverse	CATCGAATYTCAAAGCTTTGGATT	[84]
	Probe	ACKCTTGGTAGTCGTGG	
PEDV	Forward	GAAGAGGCCATCTACGATGATGT	
	Reverse	AACAGCTGTGTCCCATTCCAA	[83]
	Probe	TGTGCCATCTGATGTGACTCATGCCA	
PHEV	Forward	CCAGAAGGATGTTTATGAATTGC	
	Reverse	CCTGATGTTGATAGGCATTCA	[85]
	Probe	TGGCGCGATTAGATTTGAYAGCACACTC	
	Forward	CCAGACATGTGCCTGGTGTT	
PDCoV	Reverse	CCCYGCCTGAAAGTTGCT	[83]
	Probe	ARATGCTTTTCGCTGGCCACCTTG	
	Forward	CCAGGCCTCAAAGTGGTAAAAA	
SADS-CoV	Reverse	TGCTTACGAGCCGGTTTAGG	[83]
	Probe	ACCCAAACC/ZEN/AAGAAGCAGAGCTGTCTCAC	

## Data Availability

No new data were created or analyzed in this study. Data sharing is not applicable to this article.
